# Estimating carnivore community structures

**DOI:** 10.1038/srep41036

**Published:** 2017-01-25

**Authors:** José Jiménez, Juan Carlos Nuñez-Arjona, Carmen Rueda, Luis Mariano González, Francisco García-Domínguez, Jaime Muñoz-Igualada, José Vicente López-Bao

**Affiliations:** 1 Instituto de Investigación en Recursos Cinegéticos-(CSIC-UCLM-JCCM), Ronda de Toledo s/n.13071, Ciudad Real, Spain; 2 Tragsatec, Gerencia de Calidad, Evaluación Ambiental y Biodiversidad, C/Julián Camarillo 6B, planta 4, 28037, Madrid, Spain; 3 Subdirección General de Medio Natural. Ministerio de Agricultura, Alimentación y Medio Ambiente de España, Plaza de San Juan de la Cruz, s/n. 28075, Madrid, Spain; 4 Research Unit of Biodiversity (UO/CSIC/PA), Oviedo University, 33600, Mieres, Spain

## Abstract

Obtaining reliable estimates of the structure of carnivore communities is of paramount importance because of their ecological roles, ecosystem services and impact on biodiversity conservation, but they are still scarce. This information is key for carnivore management: to build support for and acceptance of management decisions and policies it is crucial that those decisions are based on robust and high quality information. Here, we combined camera and live-trapping surveys, as well as telemetry data, with spatially-explicit Bayesian models to show the usefulness of an integrated multi-method and multi-model approach to monitor carnivore community structures. Our methods account for imperfect detection and effectively deal with species with non-recognizable individuals. In our Mediterranean study system, the terrestrial carnivore community was dominated by red foxes (0.410 individuals/km^2^); Egyptian mongooses, feral cats and stone martens were similarly abundant (0.252, 0.249 and 0.240 individuals/km^2^, respectively), whereas badgers and common genets were the least common (0.130 and 0.087 individuals/km^2^, respectively). The precision of density estimates improved by incorporating multiple covariates, device operation, and accounting for the removal of individuals. The approach presented here has substantial implications for decision-making since it allows, for instance, the evaluation, in a standard and comparable way, of community responses to interventions.

Management goals commonly rely on information about the abundance of species. But monitoring is still one of the most controversial issues when managing wildlife^1^,^2^. Monitoring has traditionally followed a species-specific approach; although in recent times, management interventions oriented to ecosystem functioning perspectives demands reliable estimates of community-level structures, including density estimates of entire guilds^3^. Whereas obtaining reliable density estimates for some group of species, for instance, farmland birds, large herbivores in open landscapes, or soil invertebrates, seems achievable, estimates for elusive and cryptic vertebrate guilds, such as mammalian carnivores, commonly showing heterogeneous capture probabilities, remains a challenge.

The importance of obtaining reliable estimates of community structures for mammalian carnivores, including domestic species (dogs *Canis familiaris* and cats *Felis catus*), is of paramount importance to inform decision-making processes. Carnivores can be important drivers of ecosystem function, structure or dynamics. For example, this guild participates in different top-down ecosystem processes, such as trophic cascades^4^,^5^, or providing different ecosystem services, such as seed dispersal services^6^,^7^. Moreover, in the case of feral carnivores, this guild can pose particular problems for biodiversity conservation^8^,^9^.

Multiple tools have been developed over the last few decades to monitor mammalian carnivores^10^,^11^. But estimates on carnivore community structures (here we refer to the number of species present and their abundance) are still very rare^12^. Although different proxies (indices) for population abundance/density have been widely used (e.g., from sign counts and observations to number of captures in camera trapping surveys)^11^,^13^,^14^, they have also been criticised because they often do not account for variability and biases in detection probabilities among individuals^15^,^16^. Moreover, the use of non-spatially explicit analytical procedures has also been questioned because of overestimation problems in density estimates^17^. This issue has been recently overcome with the development of spatially explicit approaches^15^. However, we lack an integration of different methodological and analytical protocols to deal with different species-specific particularities yet.

Over the last decade, camera surveys have become the dominant tool to survey communities of rare and cryptic terrestrial mammals^3^,^11^,^18^,^19^. If monitoring surveys are well designed, they can yield standard and comparable data on distribution, abundance, behaviour, and community composition^20^,^21^. However, little attention has been given to the use of camera surveys to characterise community structures^3^,^19^. Within carnivore communities, although some species can be easily identified like most felid species^22^,^23^, the use of camera surveys to estimate community structures has been constrained by the fact that some individuals and species can not be identified. Moreover, only recently have detection probabilities been accounted for (in the absence of individual marks, detection rates confound abundance and detectability)^24^,^25^.

Nevertheless, the identification requirement has also been overwhelmed in recent times. State of the art analytical inference-based procedures to estimate densities using spatially explicit frameworks - linking abundance with location by estimating a latent variable representing the individual’s activity centers^26^,^27^, such as spatially explicit capture-recapture (SCR), mark-resight (SMR) and spatial count (SC) models, can be implemented even with species without marks (SMR, SC)^15^,^24^,^26^, common in several carnivore species. Interestingly, they can be combined with different sampling methods to estimate community structures. In this regard, the combination of simple camera surveys–standardisable and repeatable, and with low requirements and costs–with an integration of different spatially explicit analytical approaches (SCR, SMR, SC)^28^ –emerges therefore as a promising procedure to estimate densities for entire communities or guilds. Precision in the spatially explicit estimates, can be improved by integrating additional information, such as telemetry data from some individuals in order to improve the estimates of the movement parameters^15^,^25^, or using informative priors for sigma^24^,^29^,^30^ (i.e., the Gaussian scale parameter that determines the rate of decrease in detection probability between activity centers and traps).

In this study, we combined camera and live-trapping surveys with spatially explicit Bayesian models (SCR, SMR and SC), and additional telemetry data, to propose an integrated approach to monitor carnivore community structures, including those species where individuals can not be identified. Depending on the characteristics of each species and the number of capture events, we used SCR if all the animals were recognizable, SMR if some individuals were recognisable (including artificial and/or natural marks), and SC for unmarked animals. The procedure proposed here would allow for reliable and comparable density estimates for entire carnivore communities.

## Results

We found 9 different carnivore species in the study area (Table 1), captured 21 animals from 5 species with traps and radio-tagged (VHF) 6 individuals. Considering the encounter histories for each species, we were able to estimate the densities of red fox *(Vulpes vulpes*), stone marten (*Martes foina*), European badger *(Meles meles)*, Egyptian mongoose *(Herpestes ichneumon)*, common genet (*Genetta genetta*) and feral cat (*Felis catus*). From the list of species expected to be found in this area, we did not detect the presence of wildcat *(Felis sylvestris)*. Details on encounter frequencies for naturally and artificially marked animals as well as non-recognisable individuals for each species are shown in Table 1.

**Figure 1:**
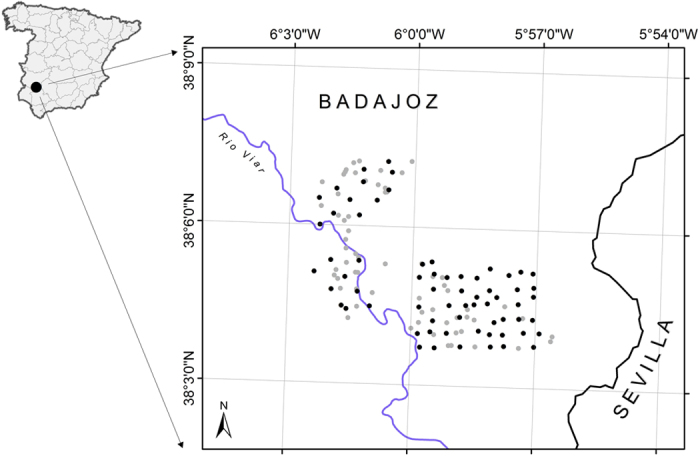
Study area showing the location of camera (black dots) and live-trapping (grey dots) devices. Mean camera spacing was 483 m. The figure was produced by José Jiménez using ArcGIS 10 (Esri Inc., Redlands, CA, USA).

The SCR approach was only used with feral cats (55 photographic events, six of which were discarded because of very poor quality images, and 11 captures with extraction), the SMR approach was used for red fox, stone martens, badgers and common genets (a total of 12 individuals trapped and marked on primary occasions or recognised, from which 8 were re-sighted on secondary occasions; and 143 capture events on secondary occasions: 48 recapture events from marked/recognisable individuals and 85 unmarked individuals), whereas the SC was used for Egyptian mongooses (77 events of unmarked individuals). We assumed that marked individuals were a random sample from the resulting state–space because marking (live-trapping) took place across the extent of the re-sighting array (cameras) (Fig. 1). For SMR models, we used different numbers of secondary occasions (Table 1) in order to maximise the number of animals marked and/or recognisable and the number of events on secondary occasions.

The model including trap-specific covariate, behavior and sampling occasions was selected for red fox (Table 2). The model with a trap-specific covariate was selected for stone martens (Table 2). The null model -with no detection probability varying between traps- was the best candidate model for feral cats (Table 2). For stone martens and feral cats, for which known removals occurred during the study period (a monitored stone marten was found dead, whereas eleven feral cats were removed from the field; Table 1), we applied the extension to account for removal of individuals in the model. However, because of the extraction of individuals we did not use covariates from individuals (behaviour) or time to adjust the baseline encounter rate. Finally, for badgers and common genets the samples of populations were too small to adjust for covariates. For Egyptian mongoose (SC) we use a trap-specific covariate (Table 2).

**Figure 2:**
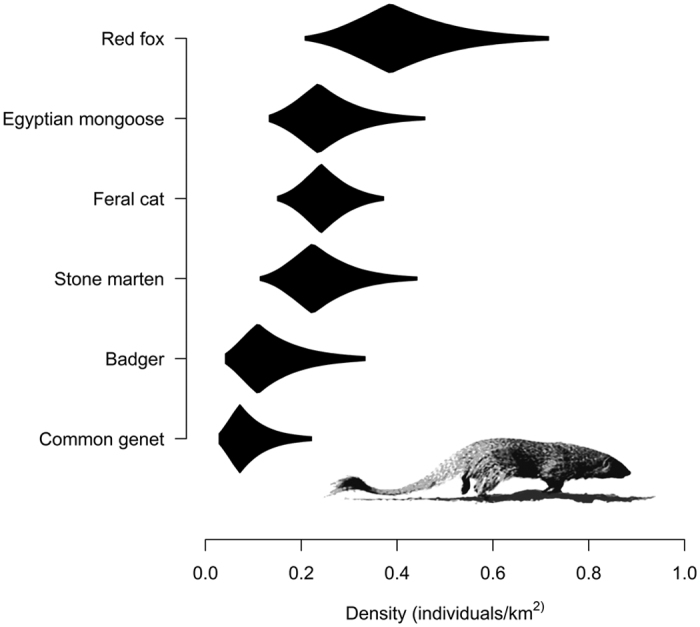
Bayesian density posterior distributions for density estimates 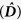 of the six carnivores analysed in this study. Polygons are shaped in proportion to the posterior probability density.

The carnivore community in the study area was dominated by red fox (density >0.4 individuals/km^2^), followed by Egyptian mongooses, feral cats, and stone martens (densities between 0.2 and 0.3 individuals/km^2^), and finally, badgers and common genets (densities <0.2 individuals/km^2^) (Fig. 2). The most abundant species were red fox, with 0.410 individuals/km^2^ (95% BCI = 0.208–0.724; CV = 0.32; Table S1). The density estimate 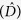 for Egyptian mongooses was 0.252 individuals/km^2^ (95% BCI = 0.125–0.450; CV = 0.33 Table S2), for feral cats 0.249 individuals/km^2^ (95% BCI = 0.150–0.376; CV = 0.24; Table S3), and 0.240 individuals/km^2^ (95% BCI = 0.114–0.442; CV = 0.35; Table S4) for stone martens. Finally, the density estimates 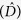 for the less abundant species were 0.130 individuals/km^2^ (95% BCI = 0.036–0.339; CV = 0.59; Table S5) for badgers and 0.087 individuals/km^2^ (95% BCI = 0.024–0.229; CV = 0.62; Table S6) for common genets.

## Discussion

Capture-recapture techniques are generally considered the “gold standard” for generating population estimates. But, in carnivore communities, the number of species and recognisable individuals are usually small. However, new analytical spatially explicit approaches allow for the estimation of densities of unmarked populations^15^,^24^,^26^, facilitating the study of carnivore community structures.

On the other hand, despite the fact that camera trapping has become popular in wildlife monitoring^28^, little attention has been paid to the use of camera surveys to characterise community structures^3^,^12^,^19^. However, this approach has been used in multispecies occupancy surveys, species richness inventories^18^,^19^ and estimates of relative abundance (indices) of species^3^,^31^, as the number of capture events has been correlated with species abundance^11^. Nonetheless, the use of indices wrongly assumes that detection probabilities are constant^15^. The combination of camera surveys, together with live-trapping (to artificially mark some animals in unrecognisable species, and a small number of individuals, e.g., 1 or 2 animals per species, to gather spatial information to be integrated into the models) and different spatially explicit analytical procedures (SCR, SMR and SC)^24^,^26^ facilitates the estimate of carnivore community structures. We recommend the use of SCR or SMR whenever possible because SC parameter estimates–when no individual information is available - are inherently less precise^15^.

**Figure 3:**
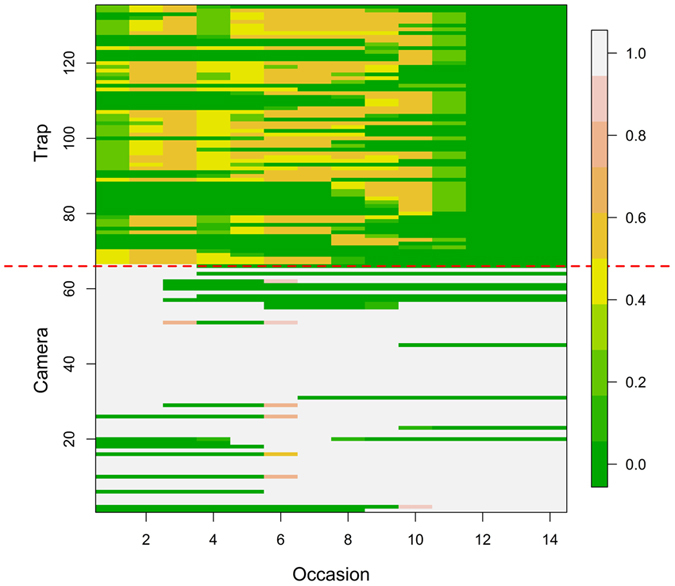
Operation of the cameras and live-trapping (trap) survey devices. The 98 sampling days are grouped into 14 7-day blocks (sampling occasions). Within each occasion, for each survey device we show operation (the number of days within each occasion where the device was operative) in a colour scale (right, 0 = the device was not operative on any day, 1 = the device was operative on all days).

We extended the use of trap operation in all models, because traps were not continuously operational during each sample occasion (Fig. 3). In addition, we accounted for removal of individuals (in our case, stone martens and feral cats). The use of covariates allowed us to identify an influence of the behavioural response in foxes to previous detection. For mongoose, red fox and stone marten there are differences between the probability of detection in traps and cameras, but not for feral cats. The numbers of captures also suggest that there are no differences for common genets. Finally, less logistical effort and lower costs are associated with the methods used here to estimate densities compared to other standard approaches. In the case of unmarked species, SMR only requires the physical capture of a few individuals, although the precision increased substantially with the proportion of marked individuals. In the case of SC, on the other hand, telemetry data could be used as data or as an informative prior for sigma^15^,^24^,^26^.

By combining spatially explicit models, we were able to estimate the structure of a close-to-complete terrestrial carnivore community in the southern Iberian Peninsula (Table 1). It is worth noting that SCR, SMR and SC density estimates are not totally comparable to non-spatially explicit procedures since the latter overestimate density estimates^17^.

With the previous caution stated, our carnivore community was dominated by red fox (Fig. 2). However, our density estimate for red fox (0.410 ± 0.133 individuals/km^2^) can be considered within the mean density values at the level of the Iberian Peninsula^32^,^33^, similar to the 0.54–0.69 red fox/km^2^ estimated using non-spatial procedures^33^. For mongooses, our density estimate (0.252 ± 0.082 individuals/km^2^) was below the estimate reported in the Doñana area, SW Spain (1.2 individuals/km^2^)^34^. The density estimates for feral cats (0.249 ± 0.059 individuals/km^2^) is the first spatially explicit density estimate for this non-native species in Spain. Additionally, to our knowledge, our density estimate for stone martens is one of the first estimates available in Spain (0.240 ± 0.083 individuals/km^2^). The density estimate for badgers was lower than those provided using telemetry and sign count data (0.23–0.67 individuals/km^2^ and 0.36–0.48 individuals/km^2^, respectively)^35^,^36^. Non-spatial capture-recapture and SCR methods have been used with common genets, showing density estimates ranging from 0.58 to 1.12 individuals/km^2 ^33,37,38 and from 0.16 to 0.79 individuals/km^2^, respectively^39^. Our density estimate (0.087 ± 0.054 individuals/km^2^) is below the estimate provided by Sarmento & Cruz (2014)^39^.

**Figure 4:**
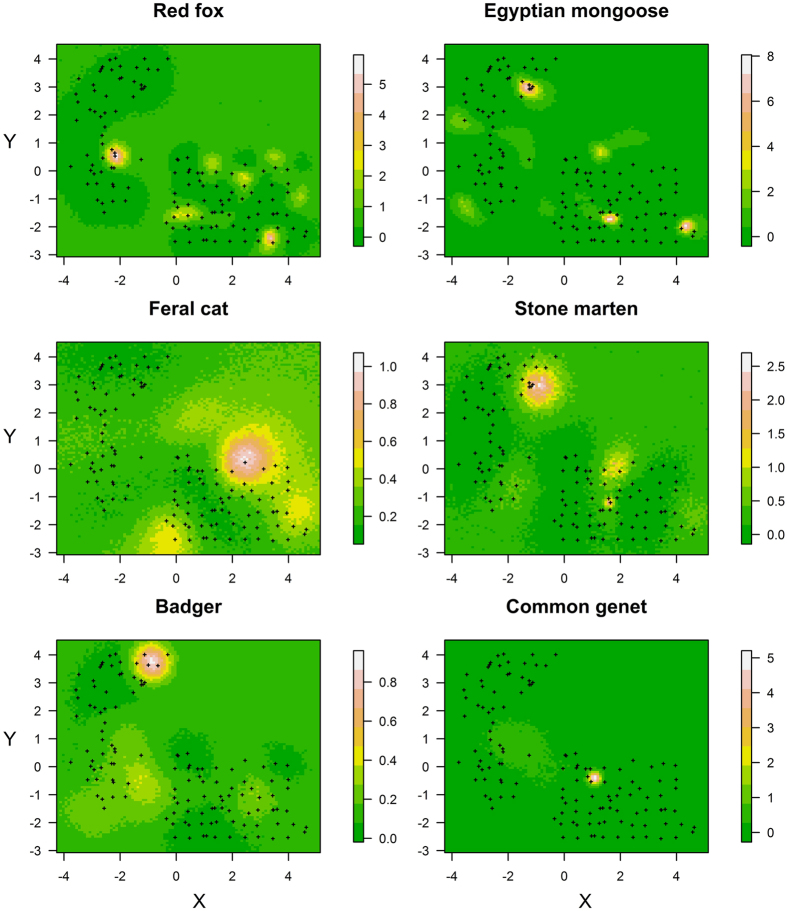
Locations of the activity centers for the six carnivores analysed in this study.

The approach used in this study additionally allowed to obtain spatial characteristics of the carnivore community, such as a comparison of the use of space among species, as the output is a set of locations of the activity centers (Fig. 4). Therefore, this approach can contribute to substantially increasing our understanding of spatial inter-specific interactions in carnivore guilds^40^,^41^.

Coefficients of variation (CV) are dependent on the models and data used. Thus, high coefficients of variation in density estimates were found in badgers (CV = 0.59) and common genets (CV = 0.62), which were those species with fewer events (8 capture events on secondary occasions for both species, and 1 and 3 marked/recognisable individuals, respectively). However, it is worth noting that even in these situations the VHF collars allowed for estimates of scale (movement) parameters^15^ and thus, their use in the model. For SC model (Egyptian mongoose with 77 events) CV was 0.33. For those species where we used SMR approaches, there were 46 and 81 capture events on secondary occasions, and 4 marked/recognisable individuals (stone marten and red fox) resulting in CV of 0.35 to 0.32, respectively. For the feral cat model using a SCR model (55 events) we found the lowest CV = 0.24.

Reliable estimates of the abundance of species and community structures are essential to inform, support and accept decision-making management processes. The management and conservation of mammalian carnivores is controversial because of the multiple socio-economic and conservation interests involved. Information about their population status or the impact of management interventions is constantly demanded not only by managers, researchers and conservationists, but also by the general public. Consequently, the quality of the data and robustness of science behind the data are crucial not only to follow an adaptive management framework, but also to build support and acceptance for management decisions and policies. Otherwise, incorrect density estimates (e.g., inflated numbers) could lead to misinterpretations of the impact of management interventions, undesirable steps in the decision-making process or can even place species at risk^42^. The rise of spatially explicit modelling approaches (SCR, SMR, SC), however, facilitates the estimation of densities and the uncertainty around estimates, for populations and communities, including those species where individuals can not be identified. We believe that integrated approaches, as the one presented here, will therefore be very useful for gathering information on community structures, and evaluating, in a standard and comparable way, changes in carnivore communities when management and conservation interventions are implemented.

## Methods

### Study area

This study was carried out in the area of Valdecigüeñas, Badajoz, SE Spain (Fig. 1), covering ca. 10,000 ha. The landscape is dominated by Mediterranean woodland, pastures and *dehesas* of holm oak (*Quercus ilex*). The area occupied by Mediterranean scrubland is residual. The Viar River crosses the study area NW-SE (Fig. 1). The main human land use in the area is sheep farming. No apex predators were present during the study period, therefore we focused on estimating the carnivore community. The main prey for carnivores in this area are European rabbits *(Oryctolagus cunniculus)*, with densities ranging between 0.5 and 1 rabbits/ha (rabbit density estimates based on pellet counts along transects)^43^. Previous to this study, but not during the sampling period, fox and Egyptian mongoose populations were controlled.

### Preliminary considerations for survey design

To estimate the structure of the carnivore community, data collection was conditioned by the analytical procedures used: SCR, SMR and SC^26^. The three methods allow for the estimation of individual’s activity centers within the prescribed state space (*S*). To do this, on the one hand, SCR relies on identifying all individuals of the population captured in the survey devices (e.g., camera-traps). On the other hand, in SMR a sample of individuals must be naturally marked or captured and tagged (or otherwise artificially marked) on the primary occasion. This must be followed by re-sighting surveys (secondary occasions) combining information from both the marked and unmarked fractions of the population. For the unmarked population, we used traps/cameras and occasion data as reduced information of “*latent*” encounter histories of individuals. Higher percentages of recognisable individuals translates into more accurate and precise parameter estimates outcomes^26^. For SC (Spatial Counts), we used only the latent encounter histories. Although the analytical method that should be prioritized is SCR, *a priori,* it is difficult to predict which model will be the most appropriate given the available data, except for the fact that SCR cannot be applied to species where individuals are not recognisable. In the case of SMR and SC models, integrating data from telemetry can improve parameter estimates^15^.

### Data collection

Between January 15, 2013 and April 23, 2013 (98 days) we collected data on the different carnivores occurring in the study area. A total of 66 cameras (models Ltl Acorn^©^ 5210, ScoutGuard^©^ SG560-8M and ScoutGuard^©^ SG570-6M) were homogeneously deployed in an area of ca. 2,300 ha (Fig. 1). Cameras were placed at a height of 30–60 cm, operated 24 h/day, and were configured with a trigger delay of 1 s. Urine from Iberian lynx *(Lynx pardinus)* was used as attractant. In Mediterranean Spain, lynx urine has been proved as one of the most effective and generalist attractants of mesocarnivores^44^. At each camera station, we impregnated a piece of cork with lynx urine and placed it on the top of a metal rod at a height of 30–60 cm set at a distance between 2.5 and 4.5 m from the camera. Cameras were checked once per month. In addition, considering the grid of cameras, we additionally placed 69 homogeneously distributed live-traps in the study area (Fig. 1). Carnivores were live-trapped using several methods: self-made box-traps (n = 14), Tomahawk^©^ box-traps (n = 33), and Collarum^®^ (n = 22). Box-traps were baited with live-prey (pigeons provided with food and water) and the Collarum with COLLARUM^®^ Canine Bait. Traps were visually checked daily early in the morning and through automatic alerts using GPS-GPRS transmitters. Sampling devices (69 traps and 66 cameras) were operative for a total (primary and secondary occasions pooled) of 1,391 and 5,395 days for traps and cameras, respectively (Fig. 3).

To set the distance between camera traps, for SCR, Sun, Fuller & Royle^45^ recommends a distance between cameras of less than 2*σ*, where *σ* is the scale or movement parameter for the target species. Chandler and Royle (2013)^26^ for SMR suggests that the distance between cameras should be enough to ensure that a given individual can potentially be captured in several traps, coercing the spatial correlation among captures. Here, as we were interested in the entire carnivore community, we used an estimate of the scale parameter 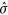
46 considering a species showing small spatial requirements. Thus, we considered acceptable a distance *d* between camera traps ranging between 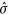 and 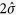, where 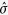 was calculated as follows:


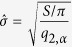


where *q*_2,*α*_ was the value of a Chi-square with 2 degrees of freedom (*α* = 0.05, *q*_2,*α*_ = 5.99) and *S* was the home range of the species (m^2^)^26^,^46^.

Based on available information on the spatial ecology of these species, we considered that *a priori* the species showing the smallest home range in our case was the Egyptian mongoose with ca. 300 ha^47^ and therefore 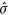 was set at 400 m. By default, we decided to use a distance between cameras of 500 m to establish the grid of camera traps. After fieldwork, the average distance between cameras was 483 m. We used a sampling area (2,300 ha) larger than the largest home range of the species expected to be present in this area and with the greatest spatial requirements: the home range for wildcats *(Felis silvestris)* (1,375 ha)^48^.

Additionally, we captured and marked a sample of individuals from each species within the study area to facilitate recognition during subsequent resighting events, a pre-requisite for SMR models (Table 1). Captured animals were marked with numbered plastic collars and photographed to facilitate their identification with the cameras. Some animals, 1–2 individuals per species, were also VHF radio-tagged (Ayama^©^). All captured animals were immobilised by intramuscular injection of medetomidine (Domitor^®^, Merial, Lyon, France) combined with ketamine (Imalgene^®^, Merial, Lyon, France). VHF collared individuals were located 2 to 3 times per week.

### Data analyses

From the camera survey data, we considered a minimum independence time interval between successive pictures of 30 min and considered those as independent events for subsequent analyses. Although we eventually checked all the pictures to identify individuals, we selected independent events automatically using *ExifTool*^49^ from R^50^ using the library *“dplyr”*^51^ and a code (Appendix S1), which allowed us to discriminate those pictures with a temporal difference >30 min. In those cases where several animals were captured in a picture, a different event was considered for each individual.

In the spatially explicit models^26^ we used the following data: i) a set of marked individuals on the primary occasions (SMR); ii) the capture histories on the secondary occasions of the individuals previously artificially marked on the primary occasions (SMR); iii) the capture histories from all individuals identifiable using natural marks (SCR and SMR); iv) trap and occasion capture histories on secondary occasions from unmarked/unidentifiable individuals (SMR and SC). Precision in the scale or movement parameter sigma (σ) was improved by integrating telemetry data, considering at least 25 locations per individual, except for badgers for which we used home range estimates in similar environments (mean home range of 975 ha, and a standard deviation that covers the home range between 475 and 1,475 ha)^52^.

Sampling occasion was defined as a sequential 7-day period. We used Poisson encounter models and data augmentation in a Bayesian framework, modified from Royle *et al*.^26^, to implement our SCR, SMR and SC models. Details on the spatially explicit Bayesian modelling approaches used are provide in Appendix S2. We modelled the influence of three covariates on density estimates by including them in the baseline encounter rate^26^. First, we included as a covariate the type of trap used (cameras *vs.* live-trapping). We included a categorical variable *tt*[*j*], which assumed the values 0 or 1 for the traps (live-trapping) and cameras, respectively. Second, we considered the local behaviour of individuals in a binary matrix *Lb*[*i, j, k*]^26^, if we had enough information from individuals (*Lb*_*ijk*_ was equal to 1 if the individual *i* was captured at least once prior to session *k*, otherwise *Lb*_*ijk*_ was set to 0). This binary matrix was used to account for differential behavioral responses of individuals to survey devices related to different capture histories, for instance, whether past detection events could influence the probability of an individual of being captured again. Finally, we considered a covariate that varied with sampling occasion (*t*[*k*]).

We modelled *λ*_*0*_ (baseline detection probability) with a log function:


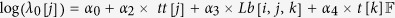


Therefore *α*_0_, *α*_2_, *α*_3_ and *α*_4_ were the parameters to be determined.

Moreover, we integrated in the models an extension to take into account known occurrences of individual removals (i.e., known mortality) with a matrix *dead*[*i, k*] indicating when (*k-*occasion) the individual (*i*) was extracted from the population. Finally, trap operation (Fig. 3) was included in all models.

The state space (*S*) is an area that includes the re-sighting grid and is sizable enough to include all individuals potentially exposed to sampling. To generate the state spaces in SMR, we used a buffer around the trap grid from the values of *σ* and *λ*_*0*_ obtained from a preliminary analysis in each case^26^.

For model selection, we used the Kuo & Mallick indicator (*w*)^53^ variable selection approach to select the best candidate model in relation to the use of different parameters in the models^26^, and we evaluated the sensitivity of posterior model probabilities to different prior specifications (normal *norm(0, 0.1*) and uniform *unif(−100, 100*)). We also used the spike and slab approach^54^. All models were run in NIMBLE^55^,^56^. We ran 3 chains of the MCMC sampler with at least 50,000 iterations in each case (see details for each species in Tables S1–S6). To check for chain convergence, we assessed MCMC convergence by visually inspecting trace plots for each monitored parameter, and we calculated the Gelman-Rubin statistic 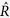
57 using the R package “*coda*”^58^ where values below 1.1 indicated convergence. For all parameters in our models, 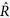 was always <1.1. Details of the models are in Appendix S3. Different informative priors for sigma could influence our results. Therefore, for red fox, Egyptian mongoose and badger, we additionally explored the influence of different priors on estimates for 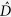 and σ (Appendix S4). For red fox and Egyptian mongoose, 95% BCI were smaller when using an informative prior for sigma compared to use a non-informative prior for sigma (Appendix S4). On the other hand, for badger, 95% BCI in 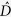 was smaller when using the informative prior for sigma (described in Appendix S3) compared to an informative prior for sigma gamma distributed (Appendix S4). We therefore selected these informative priors for sigma in our models.

### Ethics statement

All field procedures, including animal trapping, telemetry, feral cat euthanasia and camera surveys, were carried out in accordance with animal welfare regulations. Experimental protocols were approved by the Regional Government of Extremadura, Spain, under permit CN0035/13/ACA.

## Additional Information

**How to cite this article**: Jiménez, J. *et al*. Estimating carnivore community structures. *Sci. Rep.* **7**, 41036; doi: 10.1038/srep41036 (2017).

**Publisher's note:** Springer Nature remains neutral with regard to jurisdictional claims in published maps and institutional affiliations.

## Supplementary Material

Supporting Information

## Figures and Tables

**Table 1:** Summary of the raw data from the camera and live-trapping surveys used in this study, and the SCR, SMR and SC setups.

SCR setup							
	Captures x 100 cam-day	Captures x 100 traps-day	Number of occasions	Number of individuals marked or recognizable	Number of physical capture	Number of photo-captures	Number of total used events
Feral cats^1^	0.97	0.79	12	—		—	55^2^
**SMR setup**							
	**Captures x 100 cam-day**	**Captures x 100 traps-day**	**Number of secondary occasions**	**Number of individuals marked (1**^**st**^ **sampling occasions) or recognizable**	**Number of individuals resighted (2**^**nd**^ **sampling occasions)**	**Number of recaptures events (2**^**nd**^ **sampling occasions)**	**Number of unmarked events (2**^**nd**^ **sampling occasions)**
Stone marten	1.69	0	7	4	2	9	37
Red fox	4.13	0	5	4	3	32	49
Badger	0.29	0	7	1	1	3	5
Common genet	0.19	0.27	8	3	2	4	4
**SC setup**							
	**Captures x 100 cam-day**	**Captures x 100 traps-day**^1^	**Number of occasions**	**Number of individuals marked or recognizable**	**Number of physical capture**	**Number of photo-captures**	**Number of total used events**
Egyptian mongoose	1.67	0.07	12	1	1	76	77
**Other species not considered in the analyses**							
Weasel (*Mustela erminea*)	0.02						1
Otter *(Lutra lutra)*	0.02						1
European polecat (*Mustela putorious*)	0.02						1

^1^A total of 11 feral cats were extracted.

^2^Six photographs were not included because individuals could not be reliably identified.

**Table 2:** Model selection for every carnivore studied.

	Model weights comparison Kuo and Mallick (1998)		Spike and slab prior Mitchell and Beauchamp (1988)	
	*norm* (0, 0.1)	*unif* (−100, 100)	Model selected	Parameters
Red fox				
M(., ., .)	0.000	0.000		
M(*tt*, ., .)	0.000	0.0068		b1 = 1.89 ± 0.42
M(., *Lb*,.)	0.001	0.000		b2 = 3.37 ± 1.18
M(*tt*, ., *t*)	0.000	0.214		b3 = −1.13 ± 0.29
M(., *Lb, t*)	0.008	0.000		
M(*tt, Lb*,.)	0.000	0.004		
M(*tt, Lb, t*)	***0.990***	***0.713***	**x**	
Egyptian mongoose				
M(., .)	0.000	0.000		
M(*tt*,.)	***0.827***	***0.994***	**x**	b2 = 3.50 ± 0.91
M(., *t*)	0.000	0.000		b3 = 0.07 ± 0.29
M(*tt, t*)	0.173	0.006		
Stone marten				
M(.)	0.012	0.000		
M(*tt*)	***0.998***	***1.000***	**x**	b2 = 2.75 ± 1.21
Feral cats				
M(.)	***0.902***	***0.993***	**x**	
M(*tt*)	0.098	0.007		b2 = 0.00 ± 0.12

We used the Kuo & Mallick^53^ and the spike and slab^54^ approaches. The results in the first approach are post-process model weights in a comparison of all possible models. Covariates (spike and slab parameters between brackets): (i) ***Lb (b1)*** local behaviour or trap response by individual; (ii) ***tt (b2)***, a trap-specific (trap/camera) categorical covariate assuming the values of 0 or 1 for the traps and camera devices, respectively; and (iii) ***t (b3),*** a covariate that varies with sampling occasion. Selected models are denoted in bold italics or by “x”. Note that both approaches selected the same models.
