# Supplementary material for: Estimating carnivore community structures
Source: Sci Rep. 2017 Jan 25;7:41036. doi: 10.1038/srep41036 (PMC5264395; doi:10.1038/srep41036)
Supplement: Supporting Information [file srep41036-s1.pdf]

SUPPORTING INFORMATION

Estimating carnivore community structures

José Jiménez[1], Juan Carlos Nuñez Arjona[2], Carmen Rueda[2], Luis Mariano González[3], Francisco García Domínguez[3], Jaime Muñoz Igualada[2] & José Vicente López-Bao[4]

[1] Instituto de Investigación en Recursos Cinegéticos (CSIC-UCLM-JCCM); Ronda de Toledo s/n, 13071-Ciudad Real. Email: Jose.Jimenez@csic.es

[2] Tragsatec, Gerencia de Calidad, Evaluación Ambiental y Biodiversidad. C/ Julián Camarillo 6B, planta 4. 28037-Madrid. email: jcnarjona@yahoo.com (JCNA); carmen.rueda91@gmail.com (CR);jmunoz7@tragsa.es (JMI)

[3] Subdirección General de Medio Natural. Ministerio de Agricultura, Alimentación y Medio Ambiente de España. Plaza de San Juan de la Cruz s/n. 28075-Madrid. email: lmgonzalez@magrama.es(LMG); fgdominguez@magrama.es(FGD)

[4] Research Unit of Biodiversity (UO/CSIC/PA), Oviedo University, 33600-Mieres, Spain. email: jv.lopezbao@gmail.com

Appendix S1. Code (R) used to allow the selection of pictures by temporal difference. The code uses *ExifTool* in R (Harvey 2015) and the library *"dplyr"*(Wickham & Francois 2015).

```
# Install ExifTool from: http://www.sno.phy.queensu.ca/~phil/exiftool/

setwd('C:/path to your pics directory /')

system2("C:/.../exiftool", args="-common  -FNumber -DateTimeOriginal –
   FileType -csv –r C:/[your pics directory]", stdout="photodata.csv")
data <- read.csv("photodata.csv")

library(dplyr)

data$DateTimeOriginal <- as.POSIXct(strptime(data$DateTimeOriginal,
   "%Y:%m:%d %H:%M:%S"))

timestamp <- data$DateTimeOriginal

dif <- abs(difftime(timestamp, lag(timestamp))) ### We calculated temporal
                                                ### differences between pics

js <- cbind(data,dif)

write.table (js, file="js.txt")        ### Open created table and select
                                       ### temporal differences (>1800 seg)
```

References

Wickham, H. & Francois, R., 2015. dplyr: A Grammar of Data Manipulation. Available at: http://cran.r-project.org/package=dplyr.

Harvey, P., 2015. EXIFtool. Available at: http://www.sno.phy.queensu.ca/~phil/exiftool/.

Appendix S2. Details on the spatially explicit Bayesian modelling approaches used in this study.

Depending on the mesocarnivore species and data collected, in this study, we used three spatially explicit approaches: capture–recapture (SCR) models, spatial mark-resight models (SMR) and spatial counts (SC) models[1-3]. In all cases, we used Poisson encounter models and data augmentation in a Bayesian framework, modified from Royle *et al* (2014)[1].

Spatial capture-recapture (SCR) models:

This modelling approach is commonly used when all individuals in population can be individualized. SCR models postulate that the encounter probability of individuals is a decreasing function of the distance between the center of the home range (or activity center) of individual i and the location of survey devices j (s_i) (e.g., camera traps), at the occasion k. We assumed that every individual i in the population will have its own activity center s_i, and that all these activity centers will be distributed across the state space (S). The position of the survey device j is x_j and the encounter histories y, is a tri-dimensional matrix "i x j x k". In our case, the number of times that an individual i was located in a location j is Poisson distributed, with mean λ_{ijk}:

$$y \sim Poisson(\lambda_{ijk})$$

We used a Poisson distribution instead of a Binomial distribution because multiple encounters can occur in the same survey device at the same occasion k (k was defined in our case as a sequential 7-day period).

The link function between the location of survey devices and the activity centers for individuals follows a half-normal distribution[1]:

$$\lambda_{ijk} = \lambda_o exp\left(\frac{d_{ij}^2}{2\sigma^2}\right)$$

where d_{ij} is the distance between the activity center for each individual s_i and x_j, and λ_0 is the baseline encounter probability.

The total number of activity centers (*N*) is estimated by applying the augmentation data approach[1] by adding more potential individuals with all zero encounter histories, to the number of individuals re-captured. The state space *S* is generated by buffering a distance to the grid of locations of survey devices. Such buffer distance must be $>2.5\times\sigma$[1]. Density is estimated by dividing *N* by the total area of *S*.

Spatial Mark-Resight (SMR) models:

Mark-Resight approaches can be used when only a section (*m*) of a population (*N*) is naturally individualised, or artificially marked, and can be identified upon recapture, while the unmarked portion of the population ($U = N - m$) remains unidentifiable. For the section of the population that is recognisable *m*, the same encounter histories matrix *y* "i x j x k" than described above for the case of SCRs approaches is obtained. However, for the unmarked portion of the population a complementary procedure is needed. In our case, we used data from the cameras and occasions (η_{ik}) as reduced information of "latent" bi-dimensional encounter histories (traps-days) of individuals, as accumulated counts ($\eta_{ik} = \sum y_{ujk}$). In this case, encounters are assumed to be spatially correlated with the density of individual's activity centers[2]. Thus, differential exposure of individuals to detection is induced by selecting a specific distance between cameras.

For both, the marked and unmarked portions of the population, the same process is assumed. Therefore, the same parameters sigma (σ) and lambda (λ_0) (i.e., the baseline probability of encounter)[1] are used.

Every individual i has its own activity center s_i, and all activity centers are distributed across the state space (*S*). Under the SMR approach, *S* is defined as the area that includes the grid for re-sights; being sufficiently sizable to include all individuals potentially exposed to the survey. The location of the survey device j is x_j, and the encounter histories for each recognisable individual i in the camera j in the occasion k is y_{ijk}, which is an array $[i\,, j\,, k]$.

For the portion of the population that is individualised, similar to the SCR model, the number of times that an individual i is located in a camera j is Poisson distributed with a mean λ_{ijk}:

$$y \sim Poisson(\lambda_{ijk})$$

and the link function between the location of survey devices and the activity centers for individuals follows the same half-normal distribution as described before[1]:

$$\lambda_{ijk} = \lambda_o exp\left(\frac{d_{ij}^2}{2\sigma^2}\right)$$

The data model for the unmarked section of the population is also Poisson distributed, but in accumulated counts:

$$\sum_{k=1}^{K} n_{jk} \sim Poisson\left(K\lambda_0 \sum_{i=1}^{N} exp\left(d_{ij}^2/2\sigma^2\right)\right)$$

The total number of activity centers for unmarked individuals (*U*) is estimated by applying the augmentation data approach[1] and by adding more potential individuals with all zero encounter histories, to the number of individuals re-sighted *(m).*

The state space *(S)* is generated by buffering a distance function of λ_o and σ from the trap array[1,4]. Density is estimated by dividing the sum of activity centres ($m + U$) within the state space by the total area of S.

Spatial Counts (SC) models:

Under certain conditions, count data (i.e., number of encounters) are sufficient for making inferences about animal distribution and density. If the distance between neighbours' survey devices is well below the animals′ home range, it is possible to obtain spatially correlated events. Under this approach, a critical assumption is that detections are spatially correlated with the density of individual's activity centers.

All data in this model comes from unmarked individuals and therefore is based on "latent" bi-dimensional encounter histories (traps-days) of individuals. The data model for the entire unmarked population is also Poisson distributed, but in accumulated counts like in the case of SMR models. Count data (η_{ik}) are summaries of latent encounter histories. The total number of activity centers for unmarked individuals (U) is estimated by applying the augmentation data approach[1] and by adding more potential individuals with all zero encounter histories. The state space *(S)* is generated by buffering a distance function of λ_o and σ from the trap array[1,4]. Density is estimated by dividing the sum of activity centres (U) within the state space by the total area of S.

Precision in SC models is low without integrating additional information. In our case, to increase precision in parameter estimates we can use information about the home range size (e.g., extracted from the scientific literature or calculated form field information in the same study area) of animals or telemetry data (locations). In

this case, we treat this dataset separately and use telemetry data to estimate σ. Alternatively, it is possible to run joint analyses with telemetry data to estimate σ[5].

References

1. Royle, J. A., Chandler, R. B., Sollman, R. & Gardner, B. *Spatial Capture-Recapture* (Elsevier/Academic Press 2014).

2. Chandler, R. B. & Royle, J. A. Spatially-explicit models for inference about density in unmarked populations. *Ann. Appl. Stat.* **7**, 936–954 (2013).

3. Chandler, R. B. Unmarked *[Workshop-Athens GA 3/2015].* http://sites.google.com/site/spatialcapturerecapture/workshop---athens-ga-3-2015/day-4. (2015).

4. Sollmann, R., Gardner, B., Parsons, A. W., Stocking, J. J., McClintock, B. T. *et al.* A spatial mark-resight model augmented with telemetry data. *Ecology* **94**, 553–559 (2013).

5. Chandler, R. B. Unmarked Populations. *Spatial Capture-recapture. [Workshop-Athens-2016].* https://sites.google.com/site/spatialcapturerecapture/workshop-athens-2016/day4 (2016).

Appendix S3. Codes (R+NIMBLE). Modified from Royle et al. (2014).

Red Fox – SMR (behavior, trap, date) + operation + telemetry – Kuo & Mallick selection. Non-informative prior for sigma

```
code <- nimbleCode({

 psi ~ dunif(0,1)
 psim ~ dunif(0,1)
 sigma <- sqrt(1/(2*alpha1))
 alpha1 ~ dnorm(0,.1)
 alpha0 ~ dnorm(0,.1)
 alpha2 ~ dnorm(0,.1)  ## Behavior parameter
 alpha3 ~ dnorm(0,.1)  ## Trap type parameter
 alpha4 ~ dnorm(0,.1)  ## Date parameter
 w[1]~dbern(.5)  ## Kuo & Mallick parameter for behavior
 w[2]~dbern(.5)  ##,,      ,,      ,,     ,,  trap type
 w[3]~dbern(.5)  ##,,      ,,      ,,     ,,  date

 for(i in 1:bigM){
   for(k in 1:K){
     log(lam0[i,1:J,k])<- alpha0 + w[1]*alpha2*C[i,1:J,k] +w[2]*alpha3*ttrampa[1:J]+
              + w[3]*alpha4*Date[k]
   }
 }

 # Marked part
 for(i in 1:max) {
   zm[i] ~ dbern(psim)
   sm[i,1] ~ dunif(xlim[1],xlim[2])
   sm[i,2] ~ dunif(ylim[1],ylim[2])
   distm[i,1:J]<- (sm[i,1]-X[1:J,1])^2+(sm[i,2]-X[1:J,2])^2


   for(j in 1:J) {
     for(k in 1:K){
       lambdam[i,j,k]<-lam0[i,j,k]*exp(-distm[i,j]/(2*sigma^2))
       y[i,j,k]~ dpois(lambdam[i,j,k]*zm[i]*MASK[j,k])
     }
   }
 }

 # Telemetry-tagged individuals
 for(r in 1:nlocs){
   locs[r,1]~dnorm(sm[ind[r],1], 1/(sigma^2))
   locs[r,2]~dnorm(sm[ind[r],2], 1/(sigma^2))
 }

 # Unmarked part
 for(i in 1:M) {
```

```
    z[i] ~ dbern(psi)
    s[i,1] ~ dunif(xlim[1],xlim[2])
    s[i,2] ~ dunif(ylim[1],ylim[2])
    dist[i,1:J]<- (s[i,1]-X[1:J,1])^2+(s[i,2]-X[1:J,2])^2

    for(k in 1:K){
      lambda[i,1:J,k]<-lam0[i+max,1:J,k]*exp(-dist[i,1:J]/(2*sigma^2))*z[i]
    }
  }

  for(j in 1:J){
    for(k in 1:K){
      bigLambda[j,k] <- sum(lambda[1:M,j,k])
      n[j,k] ~ dpois(bigLambda[j,k]*MASK[j,k])
    }
  }
  Nu <- sum(z[1:M])
  Nm<- sum(zm[1:max])
  N<-Nu+Nm
  D<-N/area

})
```

Red Fox – SMR (behavior, trap, date) + operation – Kuo & Mallick selection. Informative prior for sigma (Chandler 2016).

Given the few recapture events for foxes in different trap locations (1 for individual "1" and 4 for individual "2"), we used an informative prior for sigma based on the home range size (95% kernel home range) from two radio-tagged individuals. The size of the home range for individual 1 (male) was 358.5 ha, whereas the home range for individual 2 (female) was 290.4 ha. We calculated the mean value (mean = 324.45 ha) and integrated this information as prior information in the model.

Following Royle et al. (2011), and assuming a Chi-squared distribution with 2 degrees of freedom, sigma is therefore given by:

sigma= sqrt(324.45*10000/pi)/sqrt(5.99) = 415.2 m (we scale by 1000: 0.415)

We used in the model log(0.415)= -0.8794768 as prior information.

```
mu.logsigma ~ dnorm(-0. 8794768, 1/(1E-06))
sd.logsigma ~ dnorm(-2.995732, 1/(1E-06))
```

# MASK: rate of operation matrix j x k representing if a device j is working at the occasion k

```
code <- nimbleCode({
 mu.logsigma ~ dnorm(-0.8794768, 1/(1E-06))
 sd.logsigma ~ dnorm(-2.995732, 1/(1E-06))
 psi ~ dunif(0,1)
 psim ~ dunif(0,1)
 alpha0 ~ dnorm(0,.1)
 alpha2 ~ dnorm(0,.1)  ## Behavior parameter
 alpha3 ~ dnorm(0,.1)  ## Trap type parameter
 alpha4 ~ dnorm(0,.1)  ## Date parameter
 w[1]~dbern(.5)  ## Kuo & Mallick parameter for behavior
 w[2]~dbern(.5)  ## ,,     ,,     ,,    ,,  trap type
 w[3]~dbern(.5)  ## ,,     ,,     ,,    ,,  date

 # Priors for spike and slab
 #for(j in 1:3){
 #  b[j]~dnorm(0,prec[j])
 #  prec[j]<-1/var[j]
 #  var[j]<-(1-gamma[j])*0.001 + gamma[j]*10
 #  gamma[j]~dbern(0.5)
 #}

 for(i in 1:bigM) {
  logsigma[i] ~ dnorm(mu.logsigma, 1/(exp(sd.logsigma)^2))
  sigma[i] <- exp(logsigma[i])
 }

 for(i in 1:bigM){
```

```
    for(k in 1:K){
      log(lam0[i,1:J,k])<- alpha0 + w[1]*alpha2*C[i,1:J,k] +
              + w[2]*alpha3*ttrampa[1:J] + w[3]*alpha4*Date[k]
    }
  }
  ## spike and slab approach
  #for(i in 1:bigM){
  #  for(k in 1:K){
  #    log(lam0[i,1:J,k])<- alpha0 + b[1]* C[i,1:J,k] +
  #            b[2]*ttrampa[1:J] + b[3]*Date[k]
  #  }
  #}

  # Marked part
  for(i in 1:max) {
    zm[i] ~ dbern(psim)
    sm[i,1] ~ dunif(xlim[1],xlim[2])
    sm[i,2] ~ dunif(ylim[1],ylim[2])
    distm[i,1:J]<- (sm[i,1]-X[1:J,1])^2+(sm[i,2]-X[1:J,2])^2
    for(j in 1:J) {
      for(k in 1:K){
        lambdam[i,j,k]<-lam0[i,j,k]*exp(-distm[i,j]/(2*sigma[i]^2))
         y[i,j,k]~ dpois(lambdam[i,j,k]*zm[i]*MASK[j,k])
      }
    }
  }

  # Unmarked part
  for(i in 1:M) {
    z[i] ~ dbern(psi)
    s[i,1] ~ dunif(xlim[1],xlim[2])
    s[i,2] ~ dunif(ylim[1],ylim[2])
    dist[i,1:J]<- (s[i,1]-X[1:J,1])^2+(s[i,2]-X[1:J,2])^2
    for(k in 1:K){
        lambda[i,1:J,k]<-lam0[i+max,1:J,k]*exp(-dist[i,1:J]/(2*sigma[i+max]^2))*z[i]
    }
  }
  for(j in 1:J){
    bigLambda[j] <- lam0*sum(lam[1:M,j])
    for(k in 1:K){
      n[j,k] ~ dpois(bigLambda[j]*MASK[j,k])
    }
  }
  Nu <- sum(z[1:M])
  Nm<- sum(zm[1:max])
  N<-Nu+Nm
  D<-N/area
})
```

Egyptian mongoose –SC (trap) + operation + telemetry – Kuo & Mallick selection. Non-informative prior for sigma. Modified from Royle et al. (2014)

```
code <- nimbleCode({

 # A priori
 psi ~ dunif(0,1)
 psi ~ dunif(0,1)
 alpha0 ~ dnorm(0,.1)
 alpha3 ~ dnorm(0,.1)   ## Trap type parameter
 alpha4 ~ dnorm(0,.1)   ## Date parameter
 sigma ~ dunif(0,5)

 w[1] ~ dbern(.5)          ## Kuo & Mallick parameter for trap type
 w[2] ~ dbern(.5)          ##    ,,        ,,         ,,         ,,          date

 for(k in 1:K){
  log(lam0[1:J,k])<- alpha0 + w[1]*alpha3*ttrampa[1:J] + w[2]*alpha4*Date[k]
 }

 for(i in 1:M) {
  z[i] ~ dbern(psi)
  s[i,1] ~ dunif(xlim[1], xlim[2])
  s[i,2] ~ dunif(ylim[1], ylim[2])
  sm[i,1] ~ dunif(xlim[1],xlim[2])
  sm[i,2] ~ dunif(ylim[1],ylim[2])
  dist[i,1:J] <- (s[i,1] - X[1:J,1])^2 + (s[i,2] - X[1:J,2])^2
  lambda[i,1:J]<-lam0[1:J]*exp(-dist[i,1:J]/(2*sigma^2))*z[i]*KT[1:J]
 }

 # Telemetry-tagged individuals
 for (r in 1:nlocs){
  locs[r,1]~dnorm(sm[1,1], 1/(sigma^2))
  locs[r,2]~dnorm(sm[1,2], 1/(sigma^2))
 }

 for(j in 1:J){
  bigLambda[j] <- sum(lambda[1:M,j])
  for(k in 1:K) {
   n[j,k] ~ dpois(bigLambda[j])
  }
 }

 N <- sum(z[1:M])
 D<-N/area

})
```

Egyptian mongoose –SC (trap) + operation + telemetry – Kuo & Mallick selection. **Informative prior for sigma** (Chandler 2016)**.**

We used an informative prior for sigma estimated from the home range size (95% kernel home range) from one radio-tagged individual (male; home range size: 380.45 ha). We used telemetry data from only one male because there is not difference in home range size between sexes (Palomares & Delibes 1991, Palomares 1994).

Following Royle et al. (2011), and assuming a Chi-squared distribution with 2 degrees of freedom, sigma is given by:

sigma= sqrt(380.45*10000/pi)/sqrt(5.99) = 449.5 m (we scale by 1000: 0.449)

We used in the model log(0.449) = -0.7994564 as prior information. Because spatial information was available only form one individual, we used SD as the log(0.05)= -2.995732.

```
mu.logsigma ~ dnorm(-0.7994564, 1/(1E-06))
sd.logsigma ~ dnorm(-2.995732, 1/(1E-06))

# MASK: rate of operation matrix j x k representing if a device j is working at the
#  occasion k

library(nimble)
## define the model
code <- nimbleCode({

  # A priori
  psi ~ dunif(0,1)
  alpha0 ~ dnorm(0,.1)
  alpha3 ~ dnorm(0,.1)   ## Trap type parameter
  alpha4 ~ dnorm(0,.1)   ## Date parameter
  mu.logsigma ~ dnorm(-0.7994564, 1/(1E-06))
  sd.logsigma ~ dnorm(-2.995732, 1/(1E-06))
  w[1] ~ dbern(.5)           ## Kuo & Mallick parameter for trap type
  w[2] ~ dbern(.5)           ## „      „       „     „  date

  for(k in 1:K){
   log(lam0[1:J,k])<- alpha0 + w[1]*alpha3*ttrampa[1:J] + w[2]*alpha4*Date[k]
  }


  for(i in 1:M) {
    z[i] ~ dbern(psi)
    s[i,1] ~ dunif(xlim[1], xlim[2])
    s[i,2] ~ dunif(ylim[1], ylim[2])
    dist[i,1:J] <- (s[i,1] - X[1:J,1])^2 + (s[i,2] - X[1:J,2])^2
    logsigma[i] ~ dnorm(mu.logsigma, 1/(exp(sd.logsigma)^2))
    sigma[i] <- exp(logsigma[i])
```

```
    for(k in 1:K){
      lambda[i,1:J,k]<-lam0[1:J,k]*
               exp(-dist[i,1:J]/(2*sigma[i]^2))*z[i]*MASK[1:J,k]
    }
  }

  for(j in 1:J){
    bigLambda[j] <- sum(lambda[1:M,j,1:K])
    n[j] ~ dpois(bigLambda[j])
  }

  N <- sum(z[1:M])
  D<-N/area

})
```

Feral cat –SCR (trap) + extraction + operation – Kuo & Mallick selection. Modified from Royle et al. (2014).

```
# MASK: rate of operation matrix j x k representing if a device j is working at the
# occasion k
# dead: matrix i x j of a binary variable representing the state of individual i in
# occasion k. An individual "i" was either alive (z[i, k] = 1) at time "t" or dead
# (z[i, k] = 0)

code <- nimbleCode({

 psi~dunif(0,1)
 alpha0 ~ dnorm(0,.1)
 alpha1 ~ dnorm(0,.1)    ## Trap type parameter
 sigma<- sqrt(1/(2*alpha1))
 alpha2 ~ dnorm(0,.1)

 w[1]~dbern(.5)              ## Kuo & Mallick parameter for trap type

 log(p0[1:J])<- alpha0 + w[1]*alpha2*ttrampa[1:J]

 for(i in 1:M){
 s[i,1] ~ dunif(xlim[1],xlim[2])
 s[i,2] ~ dunif(ylim[1],ylim[2])
 z[i]~dbern(psi)
 dist[i,1:J]<- (s[i,1] - X[1:J,1])^2 + (s[i,2] - X[1:J,2])^2
 p[i,1:J]<-  p0[1:J]*exp(-dist[i,1:J]/(2*sigma^2))*z[i]

   for(j in 1:J){
      for(k in 1:K){
       y[i,j,k]~dpois(p[i,j]*(1-dead[i,k])*MASK[j,k])
      }
    }
   }

 N<-sum(z[1:M])
 D<-N/area

})
```

Stone marten –SMR (trap) + extraction + operation + telemetry – Kuo & Mallick selection. Modified from Royle et al. (2014)

```
# MASK: rate of operation matrix j x k representing if a device j is working at the
# occasion k
# dead: matrix i x j of a binary variable representing the state of individual i in
# occasion k. An individual "i" was either alive (z[i, k] = 1) at time "t" or dead
# (z[i, k] = 0)

code <- nimbleCode({

  # A priori
  psi ~ dunif(0,1)
  psim ~ dunif(0,1)
  sigma<- sqrt(1/(2*alpha1))
  alpha0 ~ dnorm(0,0.1)
  alpha1 ~ dnorm(0,0.1)
  alpha3 ~ dnorm(0,0.1)   ## Trap type parameter

  w[1]~dbern(0.5)            ## Kuo & Mallick parameter for trap type

  log(lam0[1:J])<- alpha0 + w[1]*alpha3*ttrampa[1:J]

  # Marked part
  for(i in 1:max) {
    zm[i] ~ dbern(psim)
    sm[i,1] ~ dunif(xlim[1],xlim[2])
    sm[i,2] ~ dunif(ylim[1],ylim[2])
    distm[i,1:J]<- sqrt((sm[i,1]-X[1:J,1])^2+(sm[i,2]-X[1:J,2])^2)

    for(j in 1:J) {
      for(k in 1:K){
        lambdam[i,j,k]<-lam0[j]*exp(-distm[i,j]^2/(2*sigma^2))*(1-dead[i,k])
        y[i,j,k]~ dpois(lambdam[i,j,k]*MASK[j,k]*zm[i])
      }
    }
  }
  # Telemetry-tagged individuals
  for (r in 1:nlocs){
    locs[r,1]~dnorm(sm[1,1], 1/sigma^2)
    locs[r,2]~dnorm(sm[1,2], 1/sigma^2)
  }

  # Unmarked part
  for(i in 1:M) {
    z[i] ~ dbern(psi)
    s[i,1] ~ dunif(xlim[1],xlim[2])
    s[i,2] ~ dunif(ylim[1],ylim[2])
    dist[i,1:J]<- sqrt((s[i,1]-X[1:J,1])^2+(s[i,2]-X[1:J,2])^2)

```

```
   for(j in 1:J) {
     lam[i,j]<-lam0[j]*exp(-dist[i,j]^2/(2*sigma^2))*z[i]
   }
 }

 for(j in 1:J){
   for(k in 1:K) {
     bigLambda[j,k] <- sum(lam[1:M,j,k])
     n[j,k] ~ dpois(bigLambda[j,k]*MASK[j,k])
   }
 }
 Nu <- sum(z[1:M])
 Nm<- sum(zm[1:max])
 N<-Nu + Nm
 D<-N/area
})
```

Badger –SMR (null model) + operation. **Informative prior for sigma using a gamma distribution σ ~ Gamma(53.68, 73.26).** Modified from Chandler & Royle (2013)

```
code <- nimbleCode({

  sigma ~ dgamma(53.68, 73.26)
  psi ~ dunif(0,1)
  lam0 ~ dunif(0,5)

  # Marked part
  for(i in 1:m) {
    srec[i,1] ~ dunif(xlim[1],xlim[2])
    srec[i,2] ~ dunif(ylim[1],ylim[2])
    distmid[i,1:J]<- (srec[i,1]-X[1:J,1])^2+(srec[i,2]-X[1:J,2])^2
    lambdamid[i,1:J]<-lam0*exp(-distmid[i,1:J]/(2*sigma^2))

    for(j in 1:J) {
      yrec[i,j]~ dpois(lambdamid[i,j]*KT[j])
    }
  }

 # Unmarked part
  for(i in 1:M) {
    z[i] ~ dbern(psi)
    s[i,1] ~ dunif(xlim[1], xlim[2])
    s[i,2] ~ dunif(ylim[1], ylim[2])
    dist[i,1:J] <- (s[i,1] - X[1:J,1])^2 + (s[i,2] - X[1:J,2])^2
    lam[i,1:J] <- lam0*exp(-dist[i,1:J]/(2*sigma^2))*z[i]*KT[1:J]
  }

  for(j in 1:J){
    bigLambda[j] <- sum(lam[1:M,j])
    n[j] ~ dpois(bigLambda[j])
  }
  N <- sum(z[1:M])+ m
  D<-N/area

})
```

Badger –SMR (null model) + operation. Informative prior for sigma

We did not have spatial information form radio-tagged badgers in our study area to calculate an informative prior for sigma. However, we used information on the spatial behavior of badgers from a similar study site. We considered a home range size of 975 ha, and a SD that covered between 474 and 1475 ha (Revilla 1998).

Following Royle et al. (2014), and assuming a Chi-squared distribution with 2 degrees of freedom, sigma was therefore given by:

sigma= sqrt(975*10000/pi)/sqrt(5.99) = 719.8 m (we scale by 1000: 0.7198)

We used in the model log(0.7198)= -0.3298939 as prior information.

```
mu.logsigma ~ dnorm (-0.3298939, 1/(1E-06))
sd.logsigma ~ dnorm(-2.590267, 1/(1E-06))


# MASK: rate of operation matrix j x k representing if a device j is working at the
# occasion k

code <- nimbleCode({

  mu.logsigma ~ dnorm(-0.3298939, 1/1E-06)
  sd.logsigma ~ dnorm(-2.590267, 1/1E-06)
  psi ~ dunif(0,1)
  lam0 ~ dunif(0,5)

  for(i in 1:bigM) {
    logsigma[i] ~ dnorm(mu.logsigma, 1/(exp(sd.logsigma)^2))
    sigma[i] <- exp(logsigma[i])
  }

  # Marked part
  for(i in 1:m) {
    srec[i,1] ~ dunif(xlim[1],xlim[2])
    srec[i,2] ~ dunif(ylim[1],ylim[2])
    distmid[i,1:J]<- sqrt((srec[i,1]-X[1:J,1])^2+(srec[i,2]-X[1:J,2])^2)
    lambdamid[i,1:J]<-lam0*exp(-distmid[i,1:J]^2/(2*sigma[i]^2))

    for(j in 1:J) {
      yrec[i,j]~ dpois(lambdamid[i,j]*KT[j])
    }
  }

  # Unmarked part
  for(i in 1:M) {
    z[i] ~ dbern(psi)
    s[i,1] ~ dunif(xlim[1], xlim[2])
    s[i,2] ~ dunif(ylim[1], ylim[2])
    dist[i,1:J] <- (s[i,1] - X[1:J,1])^2 + (s[i,2] - X[1:J,2])^2
```

```
    lam[i,1:J] <- lam0*exp(-dist[i,1:J]/(2*sigma[i+m]^2))*z[i]
  }

  for(j in 1:J){
    bigLambda[j] <- sum(lam[1:M,j])

    for(k in 1:K) {
      n[j,k] ~ dpois(bigLambda[j]*MASK[j,k])
    }
  }

  N <- sum(z[1:M]) + m
  D<-N/area

})
```

Common genet –SMR (null model) + operation + telemetry. Modified from Chandler & Royle (2013)

```
# MASK: rate of operation matrix j x k representing if a device j is working at the
# occasion k
# KT<-apply(MASK,1,sum)

code <- nimbleCode({
  # A priori
  psi ~ dunif(0,1)
  psim ~ dunif(0,1)
  lam0 ~ dunif(0,5)
  sigma ~ dunif(0,5)

  # Marked part
  for(i in 1:m) {
    sm[i,1] ~ dunif(xlim[1],xlim[2])
    sm[i,2] ~ dunif(ylim[1],ylim[2])
    distm[i,1:J]<- sqrt((sm[i,1]-X[1:J,1])^2+(sm[i,2]-X[1:J,2])^2)
    lambdam[i,j]<-lam0*exp(-distm[i,j]^2/(2*sigma^2))

    for(j in 1:J) {
      yknown[i,j]~ dpois(lambdam[i,j]*KT[j])
    }
  }

  # Telemetry-tagged individuals
  for (r in 1:nlocs){
    locs[r,1]~dnorm(sm[1,1], 1/sigma^2)
    locs[r,2]~dnorm(sm[1,2], 1/sigma^2)
  }

  # Recognisable part
  for(i in 1:max) {
    zm[i] ~ dbern(psim)
    srec[i,1] ~ dunif(xlim[1],xlim[2])
    srec[i,2] ~ dunif(ylim[1],ylim[2])
    distmid[i,1:J]<- sqrt((srec[i,1]-X[1:J,1])^2+(srec[i,2]-X[1:J,2])^2)
    lambdamid[i,1:J]<-lam0*exp(-distmid[i,1:J]^2/(2*sigma^2))

    for(j in 1:J) {
      yrec[i,j]~ dpois(lambdamid[i,j]*zm[i]*KT[j])
    }
  }

  # Unmarked part
  for(i in 1:M) {
    z[i] ~ dbern(psi)
    s[i,1] ~ dunif(xlim[1],xlim[2])
    s[i,2] ~ dunif(ylim[1],ylim[2])
```

```
    dist[i,1:J]<- sqrt((s[i,1]-X[1:J,1])^2+(s[i,2]-X[1:J,2])^2)

    for(j in 1:J) {
      lambda[i,j]<-lam0*exp(-dist[i,j]^2/(2*sigma^2))*z[i]*KT[j]
     }
  }
  for(j in 1:J){
    bigLambda[j] <- sum(lambda[1:M,j])
    n[j] ~ dpois(bigLambda[j])
  }

Nm <- sum(zm[1:max])
  Nu <- sum(z[1:M])
  N <- Nm + Nu + m
  D <- N/area
})
```

References

Chandler, R.B., 2016. Unmarked Populations. *Spatial Capture-recapture. Workshop-Athens-2016*. Available at: https://sites.google.com/site/spatialcapturerecapture/workshop-athens-2016/day4 [Accessed July 30, 2016].

Chandler, R.B. & Royle, J.A., 2013. Spatially-explicit models for inference about density in unmarked populations. *The Annals of Applied Statistics*, 7(2), pp.936–954. Available at: http://projecteuclid.org/euclid.aoas/1372338474 [Accessed October 29, 2013].

Palomares, F., 1994. Site fidelity and effects of body mass on home-range size of egyptian mongooses. *Canadian Journal of Zoology*, 72(3), pp.465–469.

Palomares, F. & Delibes, M., 1991. Ecología comparada de la gineta Genetta genetta (L.) y el meloncillo Herpertes ichneumon (L.) en Doñana (SO Península Ibérica). *Boletin de la Real Sociedad Espanola de Historia Natural Seccion Biologica*, 87(1–4), pp.257–266.

Royle, J.A. et al., 2014. *Spatial capture-recapture*, Waltham, Massachusetts: Elsevier, Academic Press. Available at: http://www.sciencedirect.com/science/article/pii/B9780124059399000207 [Accessed October 9, 2013].

Appendix S4. Estimates for Density ($\widehat{D}$) and sigma (σ) parameters under different prior specifications for sigma (non-informative and informative priors), for red fox, Egyptian mongoose and Badger. Codes and explanations about sigma calculations for priors are provided in Appendix S3. BCI = Bayesian Credible Interval.

1) Red fox

   a. Non-informative prior for sigma

			BCI		
	Mean	SD	2.50%	50%	97.50%
$\widehat{D}$	0.373	0.128	0.175	0.350	0.674
sigma ($\hat{\sigma}$)	0.484	0.025	0.438	0.483	0.534

   b. Informative prior for sigma ($\hat{\sigma}$ = 0.415, prior estimated from independent telemetry data)

			BCI		
	Mean	SD	2.50%	50%	97.50%
$\widehat{D}$	0.410	0.133	0.208	0.391	0.724
sigma ($\hat{\sigma}$)	0.416	0.021	0.376	0.415	0.458

For red fox, between using a non-informative prior for sigma, or an informative prior for sigma, we observe a small difference in $\widehat{D}$ (an absolute difference of 0.037 between approaches, a difference of 3.7 individuals in 100 km^2). 95% BCI in $\widehat{D}$ were very similar between both approaches (0.499 *vs.* 0.516, for a non-informative prior for sigma and an informative prior for sigma, as described in Appendix S3, respectively; Fig. 1). In our case, for red fox, we used an informative prior for sigma because the number of spatial recaptures was very low (5 in total) and, therefore, this information was unrepresentative. Moreover, by integrating this information with telemetry data in the first procedure (a), the estimate for sigma showed a lower precision than in the second procedure (b) (0.484 *vs.* 0.416, respectively).

2) Egyptian mongoose

a. Non-informative prior for sigma

			BCI		
	Mean	SD	2.50%	50%	97.50%
$\widehat{D}$	0.306	0.109	0.142	0.291	0.566
sigma ($\hat{\sigma}$)	0.419	0.032	0.361	0.417	0.486

b. Informative prior for sigma ($\hat{\sigma}$ = 0.449, prior estimated from independent telemetry data)

			BCI		
	Mean	SD	2.50%	50%	97.50%
$\widehat{D}$	0.252	0.082	0.125	0.241	0.450
sigma ($\hat{\sigma}$)	0.450	0.022	0.407	0.449	0.488

In the case of Egyptian mongoose, we also observed a small difference in $\widehat{D}$ between both procedures (an absolute difference of 0.054, or 5.4 individuals in 100 km^2). However, in this case, 95% BCI in $\widehat{D}$ was small in the procedure using an informative prior for sigma compared to use a non-informative prior for sigma (0.325 *vs.* 0.425, respectively; Fig. 1). We used the procedure (b), an informative prior for sigma, as described in Appendix S3, because in the first procedure (a) by integrating the information from spatially correlated 77 capture events and the telemetry data, the posterior estimates for sigma showed less precision. However, by using telemetry data as informative prior, we improved the precision in the estimate of posterior of sigma (95% BCI in sigma: 0.081 *vs.* 0.125 for an informative prior for sigma and a non-informative prior for sigma, respectively).

3) Badger

a. <u>Informative prior for sigma using gamma distribution.</u> We use a less informative prior gamma distributed (see code in Appendix S3) equivalent to a home range from 390 to 2,200 ha.

			BCI		
	Mean	SD	2.50%	50%	97.50%
$\widehat{D}$	0.159	0.111	0.036	0.125	0.476
sigma ($\hat{\sigma}$)	0.722	0.103	0.532	0.718	0.936

b. <u>Informative prior for sigma</u>. Prior covering a home ranges from 474 to 1,475 ha (see code in Appendix S3).

			BCI		
	Mean	SD	2.50%	50%	97.50%
$\widehat{D}$	0.130	0.077	0.036	0.113	0.339
sigma ($\hat{\sigma}$)	0.721	0.054	0.621	0.719	0.833

Finally, for badgers, between using an informative prior for sigma gamma distributed and the informative prior for sigma described in Appendix S3, the observed differences in $\widehat{D}$ were very smaller (an absolute difference of 0.029, or 2.9 individuals in 100 km^2). This results may be due to the fact that there were not spatial recaptures, and the spatial correlation of detections was very weak because of its low number (n = 8). However, even although $\widehat{D}$ and sigma estimates were very similar, 95% BCI in $\widehat{D}$ and sigma were small in the procedure using an informative prior for sigma using log modelcompared to an informative prior for sigma gamma distributed (95% BCI $\widehat{D}$: 0.303 vs. 0.440, respectively; 95% BCI sigma: 0.212 vs. 0.404, respectively; Fig. 1). We used the procedure (b) using an informative prior for sigma as log model in order to reduce the likelihood of the Markov chain sampling unrealistic values (e.g., Ramsey et al. 2015).

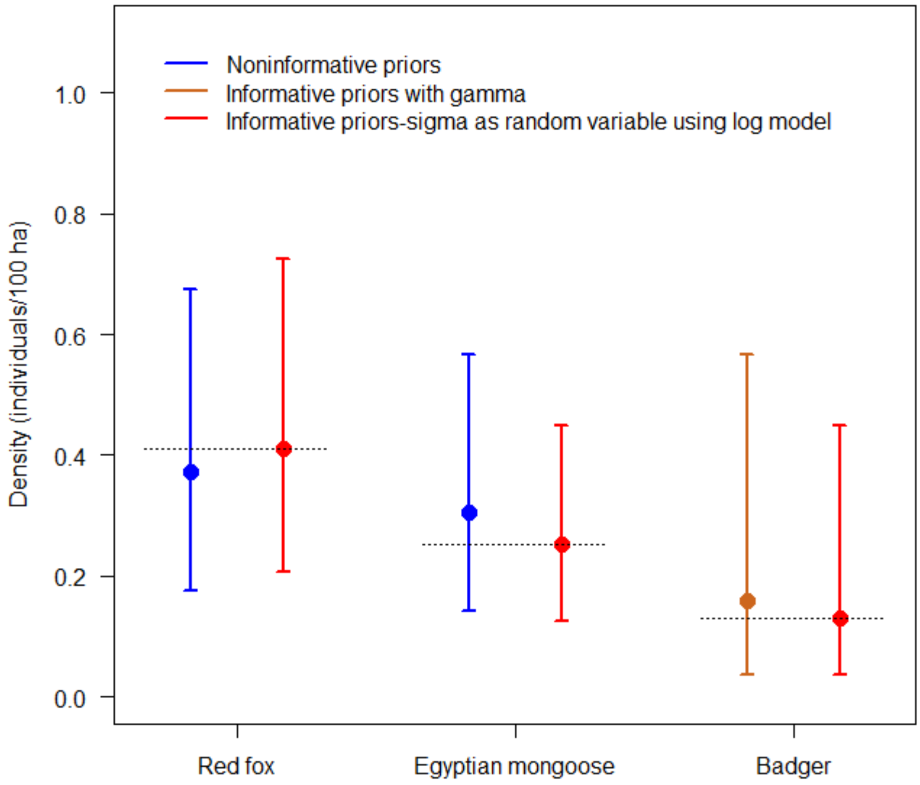

Figure 1. Posterior density estimates (mean and 95% BCI) for red fox, Egyptian mongoose and badger under different under different prior specifications for sigma.

References

Ramsey, D. S., Caley, P. A., Robley, A. Estimating population density from presence–absence data using a spatially explicit model. *J. Wildl. Manage.* ***79***, 491-499 (2015).

Table S1. Posterior summaries of parameter estimates for red fox from the selected SMR model (trap-specific covariate, behavior and sampling occasions). Estimates were based on 3 chains of 150,000 iterations and thin rate = 1, yielding 450,000 total samples from the joint posterior. BCI = Bayesian Credible Interval.

			BCI		
	Mean	SD	2.50%	50%	97.50%
$\widehat{D}$	0.410	0.133	0.208	0.391	0.724
alpha0 (α_0)	-4.709	1.269	-7.524	-4.591	-2.567
alpha2 (α_2)	1.844	0.591	1.016	1.856	2.728
alpha3 (α_3)	3.382	1.237	1.476	3.249	6.108
alpha4 (α_4)	-1.102	0.423	-1.727	-1.110	-0.546
psi (Ψ)	0.230	0.084	0.100	0.218	0.426
psim(Ψ_m)	0.789	0.169	0.378	0.830	0.993
sigma ($\hat{\sigma}$)	0.416	0.021	0.376	0.415	0.458

$\widehat{D}$: population density (individuals per square kilometer); α_0, α_2, α_3 and α_4: covariates of baseline capture probability under the Poisson encounter; α_1: parametrization of sigma; ψ: inclusion probability in the augmented data set for unmarked individuals; ψ_m: inclusion probability in the augmented data set for marked/recognisable individuals and σ: Gaussian scale parameter for the distance function.

Table S2. Posterior summaries of parameter estimates for Egyptian mongoose from the selected SMR model (trap-specific covariate). Estimates were based on 3 chains of 50,000 iterations and thin rate = 1, yielding 150,000 total samples from the joint posterior. BCI = Bayesian Credible Interval.

			BCI		
	Mean	SD	2.50%	50%	97.50%
$\widehat{D}$	0.252	0.082	0.125	0.241	0.450
alpha0(α_0)	-3.864	0.836	-5.687	-3.786	-2.449
alpha3(α_3)	3.165	0.805	1.808	3.082	4.963
psi (ψ)	0.306	0.107	0.135	0.293	0.554
sigma ($\hat{\sigma}$)	0.450	0.022	0.407	0.449	0.488

$\widehat{D}$: population density (individuals per square kilometer); α_0, and α_3: covariates of baseline capture probability under the Poisson encounter; ψ: inclusion probability in the augmented data set for unmarked individuals and σ: Gaussian scale parameter for the distance function.

Table S3. Posterior summaries of parameter estimates for feral cat from the selected SCR model (null model). Estimates were based on 3 chains of 50,000 iterations, burn-in = 10,000 iterations and thin rate = 1, yielding 120000 total samples from the joint posterior. BCI = Bayesian Credible Interval.

			BCI		
	Mean	SD	2.50%	50%	97.50%
$\widehat{D}$	0.249	0.059	0.150	0.242	0.376
alpha0(α_0)	-3.951	0.294	-4.526	-3.950	-3.387
alpha1(α_1)	0.191	0.059	0.088	0.187	0.320
alpha2(α_2)	0.009	3.002	-5.998	0.007	6.082
psi (Ψ)	0.593	0.145	0.339	0.584	0.900
sigma ($\hat{\sigma}$)	1.681	0.291	1.250	1.636	2.380

$\widehat{D}$: population density (individuals per square kilometer); α_1: parametrization of sigma; λ_0: baseline capture probability under the Poisson encounter; ψ: inclusion probability in the augmented data set for unmarked individuals and σ: Gaussian scale parameter for the distance function.

Table S4. Posterior summaries of parameter estimates for stone marten from the selected SMR model (model with trap-specific covariate). Estimates were based on 3 chains of 50,000 iterations, thin rate = 1, yielding 150,000 total samples from the joint posterior. BCI = Bayesian Credible Interval.

			BCI		
	Mean	SD	2.50%	50%	97.50%
$\hat{D}$	0.240	0.083	0.114	0.228	0.442
alpha0 (α_0)	-4.907	1.150	-7.461	-4.789	-3.003
alpha1 (α_1)	0.760	0.100	0.576	0.757	0.966
alpha2 (α_2)	2.789	1.133	0.925	2.660	5.331
psi (Ψ)	0.145	0.059	0.056	0.137	0.285
psim (Ψ_m)	0.697	0.211	0.232	0.732	0.988
sigma ($\hat{\sigma}$)	0.816	0.054	0.720	0.813	0.932

$\hat{D}$: population density (individuals per square kilometer); α_0 and α_2: covariates of baseline capture probability under the Poisson encounter; ψ: inclusion probability in the augmented data set for unmarked individuals; ψ_m: inclusion probability in the augmented data set for marked/recognisable individuals and σ: Gaussian scale parameter for the distance function

Table S5. Posterior summaries of parameter estimates for badger from the selected SMR model (null model). Estimates were based on 3 chains of 150,000 iterations, and thin rate = 1, yielding 450,000 total samples from the joint posterior. BCI = Bayesian Credible Interval.

			BCI		
	Mean	SD	2.50%	50%	97.50%
$\widehat{D}$	0.130	0.077	0.036	0.113	0.339
lam0 (λ_0)	0.057	0.044	0.013	0.047	0.163
psi (Ψ)	0.267	0.165	0.054	0.230	0.701
sigma ($\hat{\sigma}$)	0.721	0.054	0.621	0.719	0.833

$\widehat{D}$: population density (individuals per square kilometer); λ_0: baseline capture probability under the Poisson encounter; ψ: inclusion probability in the augmented data set for unmarked individuals and σ: Gaussian scale parameter for the distance function.

Table S6. Posterior summaries of parameter estimates for common genet from the selected SMR model (null model). Estimates were based on 3 chains of 50,000 iterations, and thin rate = 1, yielding 150000 total samples from the joint posterior. BCI = Bayesian Credible Interval.

			BCI		
	Mean	SD	2.50%	50%	97.50%
$\widehat{D}$	0.087	0.054	0.024	0.076	0.229
lam0 (λ_0)	0.030	0.027	0.010	0.028	0.063
psi (Ψ)	0.164	0.117	0.023	0.136	0.474
psim (Ψ_m)	0.652	0.239	0.149	0.689	0.986
sigma ($\hat{\sigma}$)	0.941	0.075	0.810	0.936	1.097

$\widehat{D}$: population density (individuals per square kilometer); λ_0: baseline capture probability; ψ: inclusion probability in the augmented data set for unmarked individuals; ψ_m: inclusion probability in the augmented data set for marked/recognisable individuals and σ: Gaussian scale parameter for the distance function.
